# Clinical evaluation of compound jinji granules in the treatment of primary premature ejaculation: a multicenter, randomized, double-blind, double-dummy, controlled trial

**DOI:** 10.3389/fphar.2026.1740832

**Published:** 2026-10-01

**Authors:** Dongyue Ma, Yun Chen, Chongfu Zhong, Qiang Han, Yong Wang, Feng Zhao, Hao Wang, Guanchao Du, Wenxiao Yu, Jun Guo, Fu Wang, Boda Guo

**Affiliations:** 1 Xiyuan Hospital of Clinical Medical College of Beijing University of Chinese Medicine, Beijing, China; 2 Department of Andrology, Xiyuan Hospital of China Academy of Chinese Medical Sciences, Beijing, China; 3 Department of Andrology, Jiangsu Province Hospital of Chinese Medicine, Nanjing, China; 4 Department of Andrology, Affiliated Hospital of Shandong University of Traditional Chinese Medicine, Jinan, China; 5 Department of Andrology, Beijing Hospital of Traditional Chinese Medicine Affiliated to Capital Medical University, Beijing, China; 6 Department of Andrology, Jining Hospital of Integrated Traditional Chinese and Western Medicine, Jining, China; 7 Department of Andrology, the First Teaching Hospital of Tianjin University of Traditional Chinese Medicine, Tianjin, China; 8 Department of Urology, Peking University Third Hospital, Beijing, China

**Keywords:** clinical trial, compound jinji granule, dapoxetine, intravaginal ejaculatory latency time, primary premature ejaculation, traditional Chinese medicine

## Abstract

**Background:**

Primary premature ejaculation (PPE) affects men’s sexual confidence, impairs couple relationships, and may lead to anxiety and depression. Compound Jinji Granule (CJG) is a traditional Chinese medicine (TCM) formula for treating PPE. Preliminary rat experiments suggested its potential in delaying ejaculation, and this study aims to further validate its clinical efficacy.

**Methods:**

This multicenter, double-blind, double-dummy, randomized controlled non-inferiority trial enrolled patients with PPE diagnosed with liver depression and kidney deficiency pattern. Participants were randomly assigned to an experimental group or a control group for 4 weeks. The experimental group received CJG plus a placebo matching dapoxetine, while the control group received a placebo matching CJG plus dapoxetine. The primary outcome was the change in stopwatch-measured average intravaginal ejaculatory latency time (IELT) during treatment. Secondary outcomes included the premature ejaculation profile (PEP), TCM symptom score, degree of IELT improvement, and clinical global impression of change (CGI-C).

**Results:**

A total of 92 subjects were enrolled, with 7 dropouts (4 in the experimental group, 3 in the control group). After 4 weeks of treatment, the average IELT in the experimental group increased from 0.29 ± 0.12 min at baseline to 2.03 ± 0.83 min (*P* < 0.01); in the control group, it increased from 0.34 ± 0.15 min to 2.48 ± 1.08 min (*P* < 0.01). Both groups showed significant improvements across all PEP outcomes. TCM symptom scores improved significantly in both groups, with the experimental group showing superior improvement compared with the control group. The proportion of patients reporting at least mild improvement was significantly higher in the experimental group than in the control group (73.91% vs. 53.33%, *P* < 0.05). In addition, no significant between-group difference was observed in the proportion of patients achieving an increase in IELT of >1 min or >3 min (*P* > 0.05). Adverse events in the experimental group included diarrhea (n = 1), abdominal bloating (n = 1), dry mouth (n = 1), and constipation (n = 1), while those in the control group included nausea (n = 4), headache (n = 3), and dizziness (n = 2). All adverse events in both groups were mild and well tolerated, and no serious adverse events were reported.

**Conclusion:**

CJG significantly prolongs IELT, improves ejaculatory control and sexual satisfaction, effectively alleviates ejaculation-related distress and couple communication, and demonstrates good safety.

## 1 Introduction

Primary premature ejaculation (PPE) is a condition involving biological, psychological, and socio-environmental factors, and has become a significant issue affecting men’s quality of life, mental health, and social relationships (Wang et al., 2024). Short intravaginal ejaculatory latency time (IELT), lack of ejaculatory control, and negative psychological impacts (such as anxiety, distress, frustration, and sexual avoidance) are considered core features of PE (Salonia et al., 2025). The reported prevalence of premature ejaculation (PE) varies widely, though most studies estimate it to be around 20%–30% (Ma et al., 2025; Sansone et al., 2024). Treatments for PPE primarily include pharmacological interventions, as well as psychological and behavioral therapies (Jannini et al., 2015). Selective serotonin reuptake inhibitors (SSRIs) are widely used for PPE and have demonstrated efficacy (Jannini et al., 2022). Dapoxetine, a short-acting SSRI, has been approved for the treatment of PE in multiple countries and regions (Zhong et al., 2022). Unlike long-acting SSRIs, which require daily administration and may take days or weeks to reach steady-state plasma concentrations, dapoxetine is rapidly absorbed and metabolized, making it more suitable for on-demand use (Sahan et al., 2020). However, existing treatments and medications still face uncertainties regarding long-term efficacy and safety, and factors such as side effects and high costs may hinder some patients from adhering to long-term therapy (Gul et al., 2022).

Compound Jinji Granule (CJG) is an empirical traditional Chinese medicine (TCM) formula used for PPE, and clinical practice in TCM has suggested its efficacy in improving ejaculatory control and prolonging IELT (Ma, Wang, Yang, et al., 2024). Although preliminary rat experiments have indicated its potential for PPE, clinical evidence remains scarce (Yu et al., 2025). Therefore, this study aims to evaluate the efficacy and safety of CJG in treating PPE through a multicenter, randomized, double-blind, double-dummy trial.

## 2 Methods

### 2.1 Study design

This was a multicenter, randomized, double-blind, double-dummy, parallel-controlled non-inferiority clinical trial designed to evaluate whether CJG are non-inferior to dapoxetine in the treatment of PPE. Eligible participants were recruited from March 2022 to September 2024 across five hospitals in China: Xiyuan Hospital of China Academy of Chinese Medical Sciences, Jiangsu Provincial Hospital of Traditional Chinese Medicine, Beijing Hospital of Traditional Chinese Medicine, Capital Medical University, Affiliated Hospital of Shandong University of Traditional Chinese Medicine, and Jining Hospital of Integrated Traditional Chinese and Western Medicine. The study was registered with the Chinese Clinical Trial Registry in 2022 (Registration No. ChiCTR2200057359).

The randomization sequence in this study was generated by an independent statistician at Xiyuan Hospital using SAS 9.4 software. This statistician was not involved in participant recruitment, screening, allocation implementation, treatment administration, outcome assessment, or data analysis. Randomization was implemented using a center-stratified, coded drug allocation approach. Based on the pre-generated randomization sequence, each participant was assigned a unique randomization number and corresponding drug code. The randomization codes and group allocation information were centrally managed by the Drug Clinical Trial Unit (GCP office) at Xiyuan Hospital and were securely maintained in sealed form, inaccessible to study personnel at the research sites. Study centers did not have access to the randomization list. Instead, investigational drugs labeled with corresponding codes were dispensed sequentially according to the order of participant enrollment, thereby ensuring allocation concealment. Prior to database lock, all study personnel—including investigators, participants, outcome assessors, and statisticians—remained blinded to treatment allocation. The randomization codes and allocation information were accessible only to the independent statistician and the clinical trial unit administrator, and unblinding was not permitted unless required in the case of a medical emergency.

All participants, along with their partners, underwent an initial screening visit. Eligible participants provided written informed consent and entered a 2-week baseline evaluation period, during which at least two sexual intercourses with IELT recordings were required. Participants with an IELT consistently shorter than 1 min during the screening period proceeded to randomization. Treatment lasted for 4 weeks, with weekly evaluations. Throughout the study, participants were required to record IELT, medication compliance, and any adverse events in a diary card.

### 2.2 Sample size calculation method

$$N=\frac{\left(1+\frac{1}{k}\right)+{{\left(\mu_{1-\alpha }+\mu_{1-\beta }\right)}^{2}\sigma }^{2}}{{\left[\left(x_{T}-x_{C}\right)-\Delta \right]}^{2}}$$

The sample size for this study was calculated based on a non-inferiority trial design, with the primary efficacy endpoint defined as the change in average IELT before and after treatment. According to previous clinical trials of dapoxetine for the treatment of PE (Buvat et al., 2009), the increase in IELT was approximately 2.3 ± 1.3 min. Based on this, CJG was assumed to have comparable efficacy to dapoxetine.

A one-sided test was applied, with the significance level (α) set at 0.025 and the type II error rate (β) set at 0.2 (corresponding to a power of 80%). The non-inferiority margin (Δ) was defined as 0.8 min. Participants were allocated to the experimental and control groups at a 1:1 ratio, and the initial sample size estimation indicated that 42 participants were required per group. To ensure study quality, loss to follow-up was strictly controlled during the trial, with a target dropout rate of less than 10%. Accordingly, the final sample size was determined to be 92 participants, with 46 participants in each group.

### 2.3 Participants

A total of 92 participants were recruited and equally assigned to the experimental and control groups (n = 46 each). All participants met the following inclusion criteria and did not meet any exclusion criteria.

Inclusion criteria: (1) Diagnosis of primary PPE according to the 2021 European Association of Urology (EAU) Guidelines on Sexual and Reproductive Health (Minhas et al., 2021). (2) Diagnosis of liver depression and kidney deficiency pattern according to TCM guidelines (Chinese Society of Andrology, 2022); (3) Disease duration >6 months; (4) Age between 23 and 50 years; (5) Sexual activity frequency ≥1 time/week, with at least six intercourses per month; (6) A stable marital relationship or a stable female sexual partner for at least 3 months before and throughout the study period; (7) Normal erectile function, defined as an International Index of Erectile Function-5 (IIEF-5) score >21; (8) Willingness to refrain from using other PE treatments during the study period (including SSRIs, herbal formulations, delay-effect condoms, or related medical devices); (9) Agreement to use effective contraception during the trial and for 3 months following the last dose.

Exclusion criteria: (1) concomitant urogenital infection; (2) history of SSRI use without therapeutic effect; (3) uncontrolled diabetes mellitus with fasting blood glucose exceeding 1.5 times the upper limit of normal; (4) history of severe cardiovascular disease; (5) severe chronic or acute hepatitis, or significant hepatic/renal impairment; (6) severe central nervous system injury within the past 6 months; (7) history of drug abuse, substance dependence, or alcoholism within the past year; (8) history of malignancy within the past 3 years; (9) allergy to study drugs or related agents (10) history of psychiatric disorders (11) participation in other clinical studies within 1 month before enrollment (12) partners with gynecological conditions affecting sexual activity or any other medical condition that precluded trial participation.

All participants were fully informed about the study procedures and provided written informed consent prior to enrollment. Participation was entirely voluntary, and participants were free to withdraw at any time without any consequences for their medical care. Given the sensitive nature of the study topic, particular attention was paid to protecting participants’ privacy and minimizing potential psychological discomfort. Assessments were conducted in a confidential setting.

### 2.4 Interventions

The experimental group received CJG, which consisted of the following botanical drugs: *Hypericum perforatum* L [Hypericaceae; *Hyperici herba*] (3 g), *Gynochthodes officinalis* (F.C.How) Razafim. and B. Bremer [Rubiaceae; *Morindae officinalis radix*] (10 g), *Paeonia lactiflora* Pall [Paeoniaceae; *Paeoniae radix alba*] (9 g), *Astragalus mongholicus* Bunge [Fabaceae; *Astragali radix*] (15 g), *Dioscorea opposita* Thunb [Dioscoreaceae; *Dioscoreae rhizoma*] (20 g), *Bupleurum chinense* DC [Apiaceae; *Bupleuri radix*] (9 g), *Acorus verus* (L.) Raf [Acoraceae; *Acori tatarinowii rhizoma*] (6 g), and *Glycyrrhiza uralensis* Fisch. ex DC [Fabaceae; *Glycyrrhizae radix et rhizoma*] (6 g). All crude drugs were authenticated and processed according to standard pharmacopoeial procedures. Participants in the experimental group took one sachet (13.33 g), dissolved in 100 mL of warm water, twice daily. In addition, they received a dapoxetine-matched placebo (starch capsule, 30 mg/tablet), taken orally 3 h before sexual intercourse. The control group received dapoxetine (30 mg/tablet), administered orally 3 h before sexual intercourse on an on-demand basis, together with a CJG-matched placebo. The CJG placebo was composed of 5% original formulation and 95% dextrin and was prepared in the same manner as the active CJG to ensure similarity in appearance, taste, and packaging.

A double-dummy design was adopted to maintain blinding, whereby all participants received both granules and tablets, with each participant receiving one active treatment (CJG or dapoxetine) along with a corresponding matched placebo. The CJG placebo contained 5% of the original formulation solely to achieve sensory matching and preserve blinding; this concentration is substantially lower than the therapeutic dose and is unlikely to produce clinically meaningful effects. CJG and its matched placebo were provided by Beijing Kangrentang Pharmaceutical Co., Ltd (Beijing, China), and were quality-controlled using high-performance liquid chromatography (HPLC) to ensure that the content of key marker compounds (e.g., hyperoside) met predefined standards (Supplementary Material 1); dapoxetine and its placebo were manufactured by Sichuan Kelun Pharmaceutical Co., Ltd.

### 2.5 Efficacy evaluation

The primary endpoint of this study was the average IELT measured by a stopwatch. The data were measured by the female partner during each sexual encounter in the baseline period and the double-blind treatment period and promptly recorded in the diary card. The weekly average IELT was calculated as the mean of the IELT values measured over the past week (for example, the average IELT for Week four was the mean of the IELT values from Week 3 to Week 4).

Secondary outcome measures included: ① changes in all dimensions of the Premature Ejaculation Profile (PEP); ② the proportion of subjects with improved average IELT, defined as average IELT >1 min and average IELT >3 min; ③ evaluation of treatment efficacy using the CGI-C scale, assessed from baseline to the end of the 4-week double-blind treatment period; ④ changes in the TCM symptoms scores.

### 2.6 Statistical analysis

This study used SAS 9.4 statistical software for data analysis. All tests were two-sided, with a significance level of 5%. Appropriate statistical methods were selected based on data types: (1) Continuous variables: parametric tests (t-test, paired t-test) or non-parametric tests (rank-sum test, paired rank-sum test, median test) were used based on normality test results. (2) Categorical variables: analyzed using χ^2^ test or Fisher’s exact test. (3) Primary efficacy analysis: changes in average IELT from baseline to Week four were compared between groups using analysis of covariance (ANCOVA) adjusted for center and baseline values, with least squares means and 95% confidence intervals calculated for between-group differences. (4) Repeated measurements: For efficacy endpoints including average IELT, PEP scores, and TCM symptom scores, a mixed model for repeated measures (MMRM) was applied. The model used the change from baseline at each visit as the dependent variable, with treatment group, visit, and the group-by-visit interaction specified as fixed effects, and baseline value included as a covariate. Subjects were treated as a random effect, and visit was specified as the repeated factor. An unstructured covariance matrix was used to model the variance–covariance structure across repeated measurements. Denominator degrees of freedom were calculated using the Kenward–Roger method. The model was fitted using all available data, with parameters estimated by restricted maximum likelihood under the missing-at-random assumption. Least-squares mean differences between groups at each visit and their 95% confidence intervals were estimated, and multiplicity was controlled using the Tukey adjustment to control the overall type I error rate. (5) Analysis populations: all randomized subjects who received the study drug were included in the Full Analysis Set (FAS), which included all treated patients with baseline measurements and at least one post-baseline measurement during treatment, used for baseline characteristic analysis and efficacy evaluation.

## 3 Results

### 3.1 Analysis of general data

Of the 126 participants screened, 92 were enrolled and randomized to the experimental group (n = 46) or the control group (n = 46). During the study, 7 participants discontinued the trial (Figure 1). In the experimental group (n = 4), all withdrew voluntarily due to personal time conflicts and decreased treatment willingness. In the control group (n = 3), one was lost to follow-up due to ineffective contact, and two withdrew voluntarily due to lack of confidence in efficacy and family commitments. No significant differences in demographic or baseline clinical characteristics were observed between the two groups prior to the intervention (*P* > 0.05) (Table 1).

**FIGURE 1:**
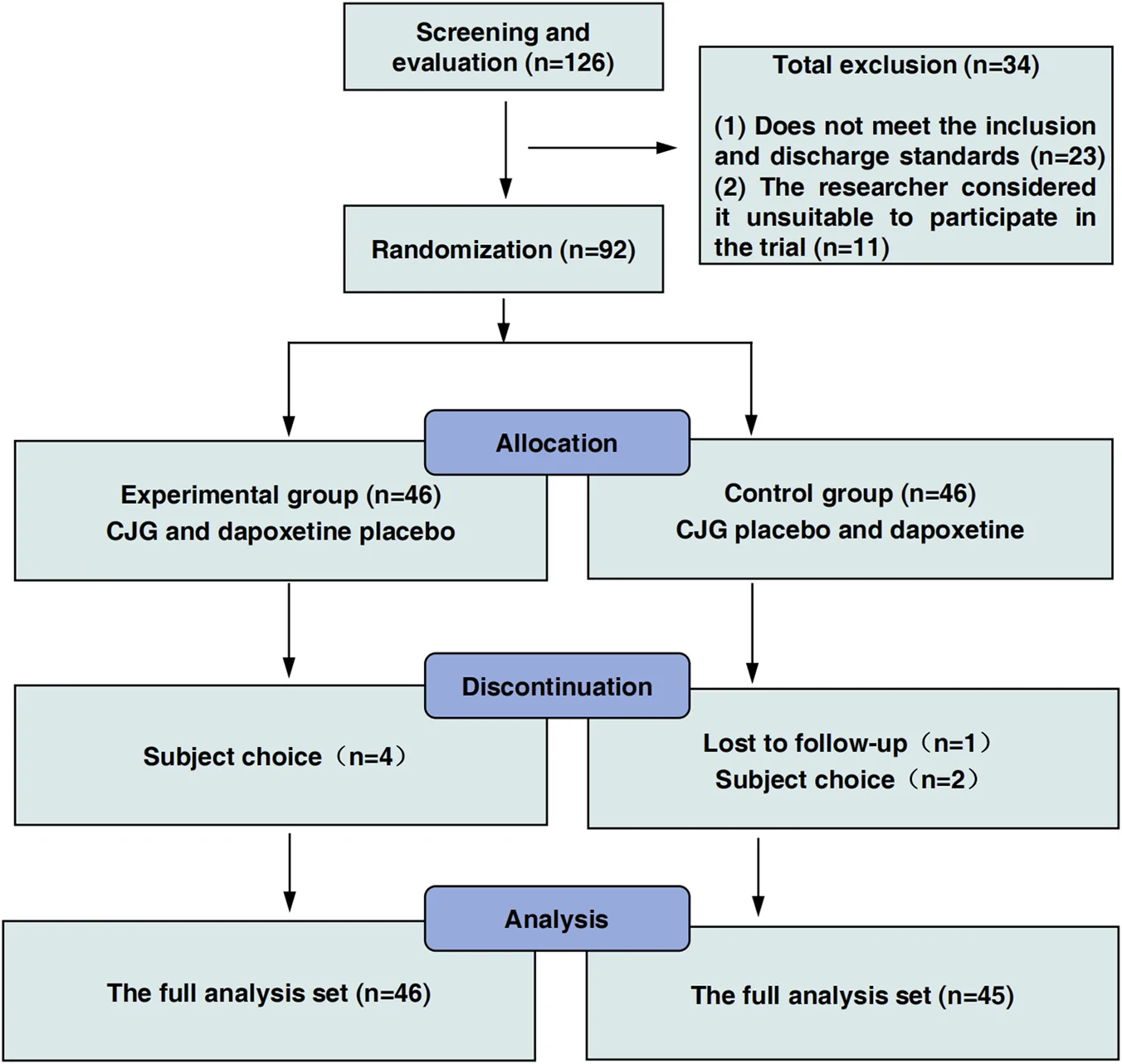
Flowchart of subject recruitment. CJG, Compound Jinji Granule.

**TABLE 1:** Baseline demographic and clinical characteristics.

Characteristic	Experimental group (n = 46)	Control group (n = 45)	*P*
Age (years)	34.98 ± 6.39	33.13 ± 7.36	0.20
BMI (kg/m^2^)	23.59 ± 2.28	22.73 ± 2.53	0.09
Disease duration (years)	7.98 ± 5.57	9.22 ± 7.18	0.36
IIEF-5 score	23.42 ± 1.31	23.66 ± 0.91	0.32
Education level (%)			
College or above High school or below	34 (73.91%) 12 (26.09%)	31 (68.88%) 14 (31.11%)	0.60
Smoking (%)			
Yes No	15 (32.61%) 31 (67.39%)	13 (28.89%) 32 (71.11%)	0.15
Alcohol use (%)			
Yes No	8 (17.39%) 38 (82.61%)	10 (22.22%) 35 (77.78%)	0.56
Marital status (%)			
Married Unmarried	40 (86.96%) 6 (13.04%)	38 (84.44%) 7 (15.56%)	0.73
Sexual frequency (%)			
1–2 times/week ≥3 times/week	41 (89.13%) 5 (10.87%)	42 (93.33%) 3 (6.67%)	0.48

IIEF-5, International Index of Erectile Function Questionnaire-5.

### 3.2 Efficacy analysis

#### 3.2.1 IELT

As IELT data were approximately normally distributed, parametric tests were applied. IELT was significantly prolonged in both the experimental and control groups within 1 week, and the effect persisted until week 4 (Figure 2). Baseline IELT was comparable between groups. After 4 weeks of double-blind treatment, IELT increased significantly in both groups (Table 2). The differences in average IELT before and after the 4-week treatment were 1.74 ± 0.75 min for the experimental group and 2.14 ± 1.07 min for the control group, with a statistically significant difference between the groups. Analysis of covariance indicated that the efficacy was primarily influenced by the treatment group and baseline IELT levels, while the study center had no significant impact on the results (*P* > 0.05).

**FIGURE 2:**
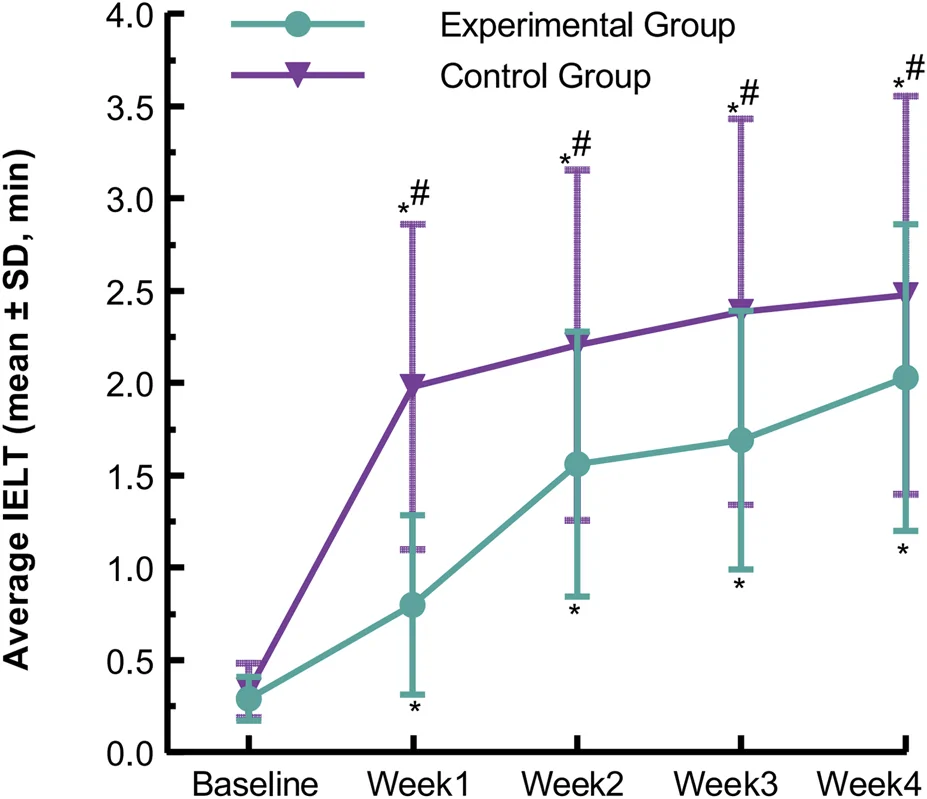
Comparison of Changes in Average IELT. Note: *P < 0.05 compared with the same group before treatment; #*P* < 0.05 compared with the control group at the same time point.

**TABLE 2:** Changes in IELT and PEP-related outcomes over time and between-group comparisons.

Indicators	Group	Baseline	1 Week	2 Week	3 Week	4 Week	Between-group difference (Week 4-baseline)	Group		Group × time interaction	
								*F*	*P*	*F*	*P*
Average IELT (min)	Experimental	0.29 ± 0.12	0.80 ± 0.49^ab^	1.56 ± 0.72^ab^	1.69 ± 0.70^ab^	2.03 ± 0.83^ab^	0.40 [-0.79, −0.02]^b^	19.841	0.000	20.536	0.000
	Control	0.34 ± 0.15	1.98 ± 0.88^a^	2.21 ± 0.95^a^	2.39 ± 1.04^a^	2.48 ± 1.08^a^					
PEP scores	Experimental	4.80 ± 2.10	6.35 ± 2.52^a^	7.00 ± 2.63^a^	7.57 ± 2.67^a^	8.30 ± 2.58^a^	0.54 [-1.47, 0.38]^c^	0.008	0.930	0.638	0.637
	Control	4.47 ± 2.11	6.51 ± 2.25^a^	7.07 ± 2.49^a^	7.67 ± 2.50^a^	8.51 ± 2.23^a^					
Perceived control over ejaculation	Experimental	0.57 ± 0.62	0.91 ± 0.69^ab^	1.13 ± 0.72^ab^	1.30 ± 0.79^ab^	1.48 ± 0.84^ab^	0.40 [-0.70,0.10]^b^	18.923	0.000	4.243	0.004
	Control	0.73 ± 0.58	1.60 ± 0.62^a^	1.78 ± 0.70^a^	1.91 ± 0.67^a^	2.04 ± 0.64^a^					
Ejaculation-related personal distress	Experimental	1.28 ± 1.00	1.80 ± 0.98^a^	1.96 ± 0.97^a^	2.13 ± 0.96^a^	2.28 ± 0.86^a^	0.13 [-0.49,0.23]^c^	1.876	0.174	0.421	0.793
	Control	1.16 ± 0.95	1.51 ± 0.92^a^	1.64 ± 0.88^a^	1.87 ± 0.94^a^	2.09 ± 0.82^a^					
Satisfaction with sexual intercourse	Experimental	1.13 ± 0.81	1.61 ± 0.80^a^	1.70 ± 0.89^a^	1.83 ± 0.93^a^	2.07 ± 0.83^a^	0.07 [-0.25,0.39]^c^	1.479	0.227	0.425	0.790
	Control	0.84 ± 0.67	1.42 ± 0.62^a^	1.60 ± 0.69^a^	1.71 ± 0.70^a^	1.91 ± 0.70^a^					
Ejaculation-related interpersonal difficulty	Experimental	1.83 ± 1.00	2.02 ± 1.11	2.22 ± 1.05^a^	2.30 ± 0.99^a^	2.48 ± 0.96^a^	0.08 [-0.42,0.25]^c^	0.182	0.671	1.032	0.396
	Control	1.73 ± 1.20	1.98 ± 1.12	2.04 ± 1.15^a^	2.18 ± 1.11^a^	2.47 ± 1.08^a^					

Data are presented as mean ± SD, or mean difference [95% CI]. IELT, intravaginal ejaculatory latency time; PEP, premature ejaculation profile. Note: a: compared with before treatment in the same group, *P* < 0.05; b: compared with the control group during the same period, *P* < 0.05; c: compared with the control group during the same period, *P* > 0.05.

#### 3.2.2 PEP scores

This study referred to previous clinical research and used the PEP scale for assessment (McMahon et al., 2010). The scale encompasses four dimensions: perceived control over ejaculation (PCOE), satisfaction with sexual intercourse (SSI), ejaculation-related personal distress (ERPD), and ejaculation-related interpersonal difficulty (ERID). The baseline characteristics of the two groups were comparable (Table 2). The results showed that subjects in both groups treated with CJG or 30 mg dapoxetine exhibited improvements in PEP outcome scores.

In terms of improving PCOE, both groups showed significantly higher scores at each follow-up visit compared to baseline, with the most notable improvement observed after 4 weeks of treatment (Figure 3A). However, the control group demonstrated higher scores than the experimental group at the 4-week timepoint (*P* < 0.05). After 4 weeks of treatment, both groups exhibited significant improvements in scores related to ERPD, SSI, and ERID compared to baseline (Figures 3B–D). However, no statistically significant differences were observed between the two groups at the same time points (*P* > 0.05).

**FIGURE 3:**
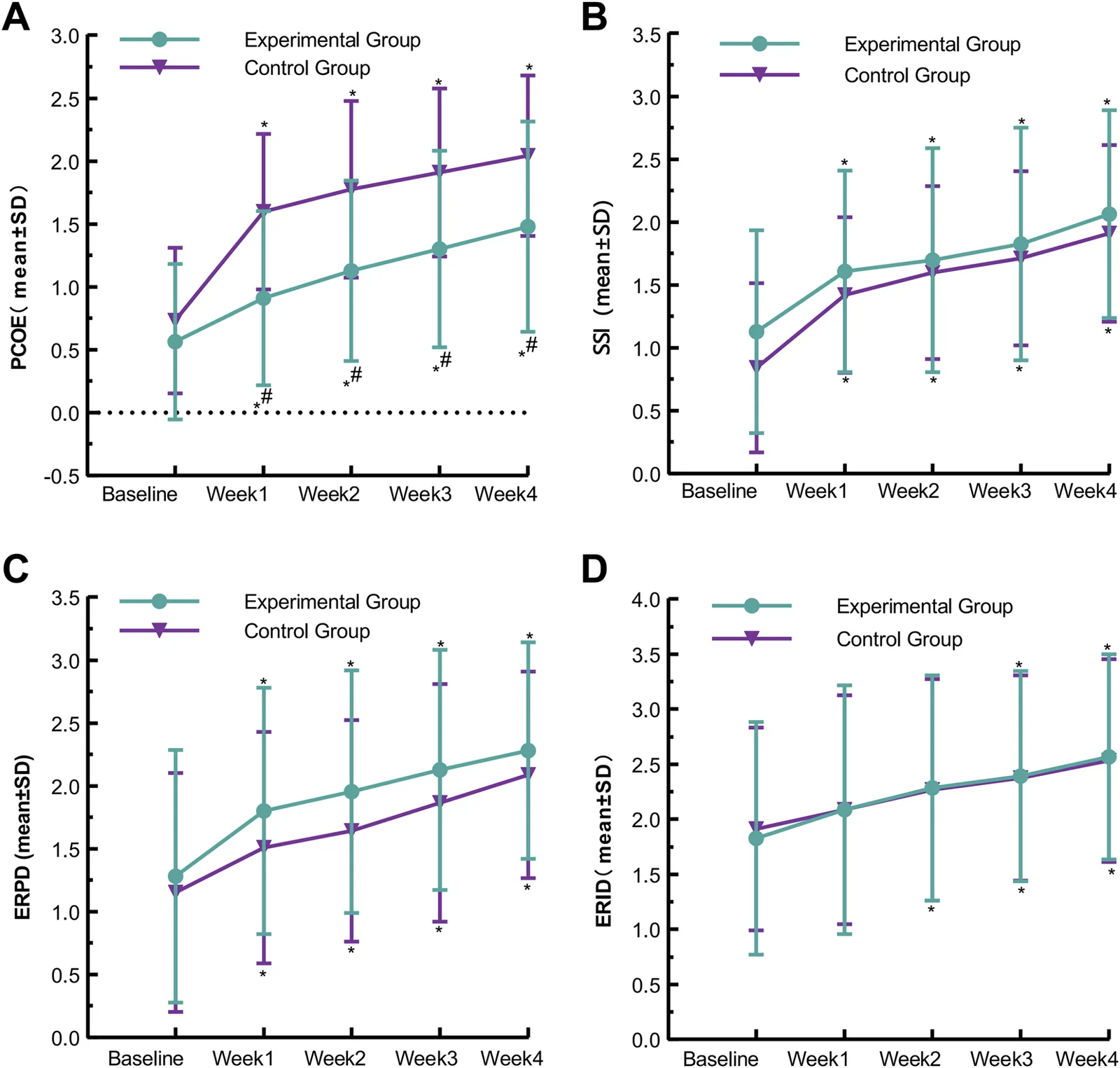
Improvement in PEP Scores Between the Two Groups. **(A)** Changes in PCOE. **(B)** Changes in ERPD. **(C)** Changes in SSI. **(D)** Changes in ERID. Note: PCOE: perceived control over ejaculation; ERPD: ejaculation-related personal distress; SSI: satisfaction with sexual intercourse; ERID: ejaculation-related interpersonal difficulty. Note: *P < 0.05 compared with the same group before treatment; #*P* < 0.05 compared with the control group at the same time point.

#### 3.2.3 Comparison of IELT improvement and CGI-C scores

After 4 weeks of treatment, no significant differences were observed between the two groups in terms of the degree of improvement in IELT (Table 3): 89.13% of patients in the experimental group achieved an IELT >1 min (compared to 82.22% in the control group), while 13.10% achieved an IELT >3 min (compared to 24.44% in the control group).

**TABLE 3:** Comparison of IELT improvement and CGI-C efficacy.

Outcome measure	Experimental group (N = 46)	Control group (N = 45)	*P*
>1 min N (%)	41 (89.13%)	37 (82.22%)	0.35
>3 min N (%)	6 (13.10%)	11 (24.44%)	0.16
At least “slightly better” CGI rating, N (%)	34 (73.91%)	24 (53.33%)	0.04
“Better” or “much better” CGI rating, n (%)	9 (19.57%)	12 (26.67%)	0.41

Data are presented as n (%). IELT, intravaginal ejaculatory latency time. “>1 min” and “>3 min” indicate post-treatment IELT, thresholds; CGI-C, clinical global impression of change: “at least slightly better” = scores 1–3; “better or much better” = scores 2–3. *P* values were calculated using the chi-square test or Fisher’s exact test; *P* < 0.05 was considered statistically significant.

Regarding CGI-C scores, 73.91% of patients in the experimental group reported at least “slightly better” improvement, a proportion significantly higher than that of the control group. However, at the higher improvement levels of “better” or “much better,” the proportion in the experimental group (19.57%) showed no statistically significant difference compared to the control group (Table 3).

### 3.3 Safety analysis

A total of 91 participants were included in the safety analysis, with 46 in the experimental group and 45 in the control group (Table 4). The overall incidence of adverse events was 8.7% (4/46) in the experimental group and 20.0% (9/45) in the control group. In the experimental group, adverse events were mainly mild gastrointestinal symptoms, including diarrhea, abdominal bloating, dry mouth, and constipation, each occurring in 1 case (2.17%). In the control group, adverse events included nausea (4 cases, 8.89%), headache (3 cases, 6.67%), and dizziness (2 cases, 4.44%). Overall, adverse events in both groups were mild and tolerable, with no serious adverse events reported.

**TABLE 4:** Incidence of adverse events.

Adverse events	Experimental group (n = 46)	Control group (n = 45)
Any adverse event	4 (8.70%)	9 (20.00%)
Diarrhea	1 (2.17%)	0
Abdominal bloating	1 (2.17%)	0
Dry mouth	1 (2.17%)	0
Constipation	1 (2.17%)	0
Headache	0	3 (6.67%)
Nausea	0	4 (8.89%)
Dizziness	0	2 (4.44%)

## 4 Discussion

From the perspective of TCM, PPE is closely associated with functional imbalances of the brain, heart, kidneys, and the male reproductive system, the latter being referred to as the “essence chamber” (Jing Shi) in TCM terminology (Zhao et al., 2024). Professor Guo Jun proposed a theoretical framework and therapeutic approach for PE based on the “brain–heart–kidney–essence chamber axis” (Guo, 2021). Guided by this theory, CJG was developed for the treatment of PPE (Figure 4).

**FIGURE 4:**
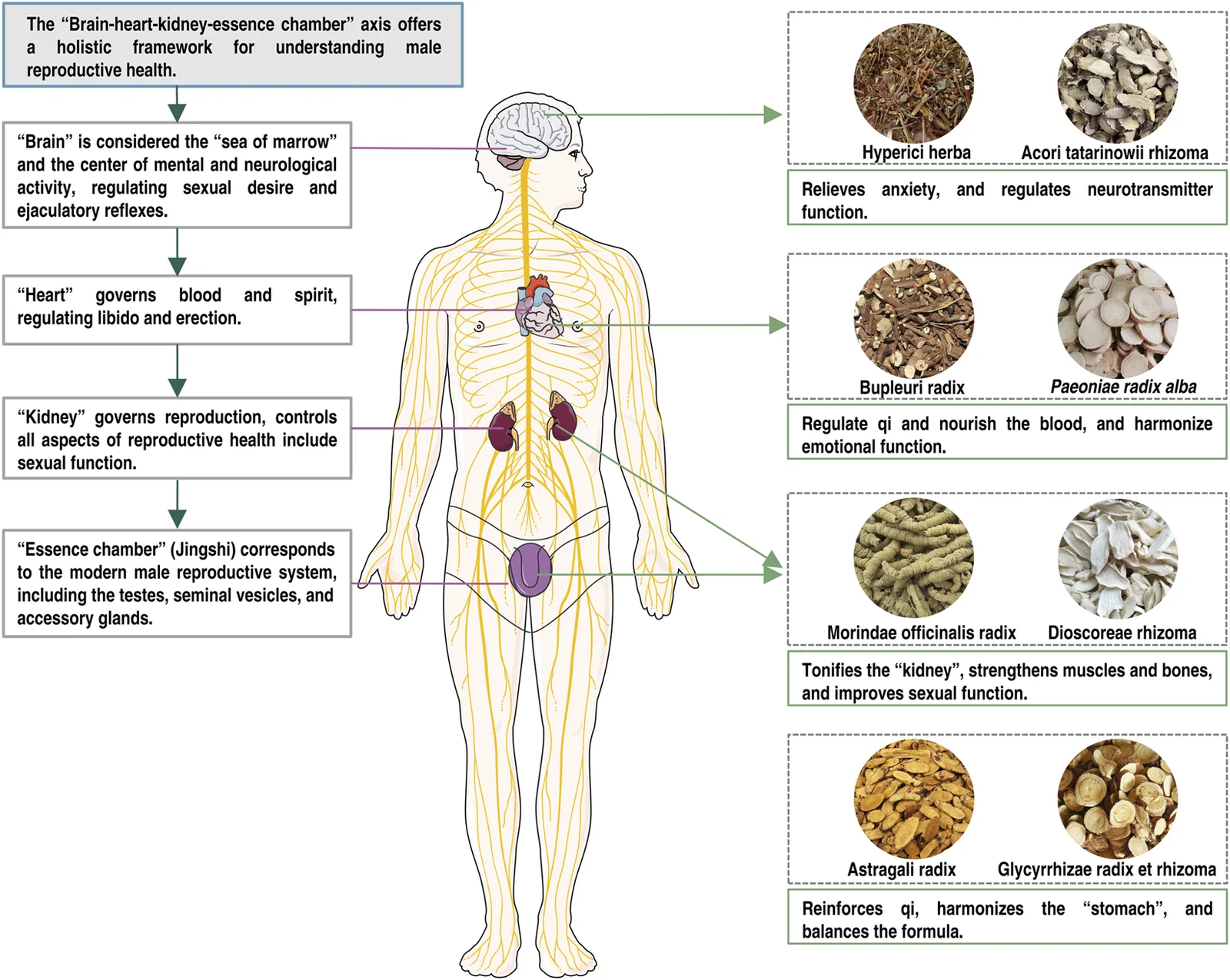
Schematic diagram of CJG for the treatment of PPE under the guidance of the “brain–heart–kidney–essence chamber axis.” This axis is a concept derived from TCM theory and represents one of the key frameworks guiding medication use in TCM andrology, differing from the organ-based concepts in modern medicine. The botanical drugs shown in the figure constitute the components of CJG.

In recent years, TCM compound formulations have shown great potential in improving ejaculatory function (Xu et al., 2024; Qian et al., 2025). Guo et al. found that, compared to a placebo, the group treated with Qiaoshao Formula exhibited prolonged IELT, reduced PE diagnostic tool scores, improved sexual satisfaction, and no adverse reactions after 4 weeks of treatment (Guo et al., 2016). Moreover, the combination therapy group (Qiaoshao Formula + dapoxetine) demonstrated advantages over dapoxetine alone (Guo et al., 2021). Han et al. discovered that Shugan Yidan Formula increased the expression of hypothalamic dopamine transporter (DAT) and decreased the expression of D2/D3 receptors in a PE rat model induced by a DA autoreceptor agonist, thereby alleviating PE symptoms (Han et al., 2023). In previous animal experiments, our team observed that CJG significantly improved sexual behavior in a PE rat model, prolonged ejaculation latency, and reduced ejaculation frequency (Yu et al., 2025).

From a methodological perspective, IELT was selected as the primary endpoint, in line with the core outcome framework for PPE research. However, IELT has recognized limitations, including limited applicability across different sexual contexts and susceptibility to confounding by age, sexual experience, and psychological state. To enhance cohort homogeneity, participants were enrolled based on IELT <1 min in combination with additional inclusion criteria, thereby balancing objectivity with clinical feasibility. In addition, a placebo containing 5% of the active formulation was used to maintain blinding. Although this dose is substantially lower than the therapeutic range, it may theoretically bias results toward the null, potentially leading to an underestimation of the true treatment effect.

This study shows that CJG significantly prolongs IELT in patients with PPE, while also improving quality of sexual life and reducing psychological burden. Although dapoxetine demonstrated numerically greater effects in prolonging IELT and enhancing ejaculatory control, there were no statistically significant between-group differences in the proportions of patients achieving clinically meaningful thresholds (IELT >1 min and >3 min), and overall efficacy (PEP total score) was comparable.

With respect to patient-reported outcomes, CJG performed comparably to dapoxetine in alleviating emotional distress and improving partner communication, and a higher proportion of patients in the experimental group reported subjective improvement on the CGI-C (self-assessed under blinded conditions). Notably, this subjective benefit was not entirely consistent with between-group differences in objective measures (IELT and ejaculatory control), indicating that perceived treatment benefit is not solely determined by physiological outcomes but is also influenced by tolerability and overall treatment experience. The lower incidence of adverse events in the experimental group may have contributed to higher patient satisfaction. In addition, CJG is characterized by its effects of “soothing the liver and tonifying the kidney,” which may modulate emotional states and related symptom patterns (Supplementary Material 1), thereby influencing neuropsychological processes and overall patient perception (Ma, Wang, Wang, et al., 2024).

In terms of safety, CJG was generally well tolerated, with a lower incidence of adverse events than dapoxetine; these were predominantly mild and transient gastrointestinal symptoms. By contrast, adverse events such as headache and dizziness were more frequently reported in the control group, consistent with its serotonergic pharmacological profile (Buvat et al., 2009). According to the Chinese Pharmacopoeia (2020 Edition, Part I), the individual herbal components of CJG have a favorable safety profile at conventional dosages. Nevertheless, as a compound formulation, its long-term safety and potential drug–drug interactions require further evaluation in studies with larger sample sizes and extended follow-up.

Several limitations should be acknowledged. First, the relatively small sample size and short treatment duration limit the assessment of sustained efficacy and long-term safety. Second, the study population was restricted to patients with PPE corresponding to the TCM pattern of liver depression and kidney deficiency, which may limit generalizability. Future studies are warranted to further validate the efficacy and safety of CJG. Integration of neuroimaging, reproductive hormone assays, and neurotransmitter analyses may help elucidate its underlying mechanisms. In addition, exploring the role of CJG in combination treatment strategies may contribute to optimizing individualized management of PPE.

## 5 Conclusion

In this multicenter randomized controlled trial, CJG demonstrated clinical efficacy in men with PPE by prolonging IELT, alleviating distress, and improving sexual satisfaction. CJG produced clinically meaningful improvements with fewer typical dapoxetine-related adverse events, and may be a candidate option for patients who cannot tolerate on-demand SSRIs, pending larger and longer trials.

## Data Availability

The raw data supporting the conclusions of this article will be made available by the authors, without undue reservation.
