# Supplementary material for: Clinical evaluation of compound jinji granules in the treatment of primary premature ejaculation: a multicenter, randomized, double-blind, double-dummy, controlled trial
Source: Front Pharmacol. 2026 Oct 1;17:1740832. doi: 10.3389/fphar.2026.1740832 (PMC13669152; doi:10.3389/fphar.2026.1740832)
Supplement: Supplementary file 1 [file Supplementaryfile1.docx]

**Additional file 1**

**1 Definition of liver depression and kidney deficiency syndrome**

**Basis of traditional Chinese medicine (TCM) syndrome differentiation:**

(Refer to "Multidisciplinary Guidelines for Integrated Chinese and Western Medicine Diagnosis and Treatment of Premature Ejaculation (2021 Edition)" by the Andrology Branch of China Information Association of Traditional Chinese Medicine)

**Primary symptoms:** Chest and flank distension pain, Soreness and weakness of waist and knees.

**Secondary symptoms:** depression, dizziness and tinnitus, frequent sighing, indifferent libido.

**Tongue and pulse:** Pale dark tongue, wiry and thready pulse.

Possessing 1 primary symptom and 2 secondary symptoms, and conforming to the tongue and pulse, it can be recognized as damp-heat syndrome.

Table S1. TCM syndrome scale score.

|  | Symptom | Gauge | Score | Grading system |
| --- | --- | --- | --- | --- |
| Primary  symptom | Chest and flank distension pain | Degree |  | 0 score: None 2 score: Mild pain, does not affect work and life 4 score: Moderate pain, affects work and life partly 6 score: Severe pain, affects work and life fully |
|  | soreness and weakness of the waist and knees | Degree |  | 0 Score: None 2 Score: Mild weakness, does not affect work and life 4 Score: Moderate weakness , affects work and life partly 6 Score: Severe weakness, affects work and life fully |
| Secondary symptoms | Depression | Frequency |  | 0 score: None 1 score: Occasionally 2 score: Sometimes 3 score: Often |
|  | Dizziness tinnitus | Frequency |  | 0 score: None 1 score: Occasionally 2 score: Sometimes 3 score: Often |
|  | Frequent sighing | Frequency |  | 0 score: None 1 score: Occasionally 2 score: Sometimes 3 score: Often |
|  | indifferent libido | Frequency |  | 0 score: None 1 score: Occasionally 2 score: Sometimes 3 score: Often |
| Total symptom score |  | |  | Total symptom score = total primary symptom scores + total secondary symptom scores. |

**Additional file 2**

The dose of each herb of Compound Jinji Granules (CJG) in different groups and the detailed description of drug quality control and safety experiments.

Table S2. The dose of each botanical drug of CJG in different groups in the Study.

| No. | Latin Name | Family | Medicinal Part | Dosage (g) | Pharmacopoeia Reference |
| --- | --- | --- | --- | --- | --- |
| 1 | Hypericum perforatum L. | Hypericaceae | Aerial parts | 3 | Chinese Pharmacopoeia (2020 Edition, Part I), p. 242 |
| 2 | *Gynochthodes officinalis (F.C.How)* Razafim. & B.Bremer | Rubiaceae | Root | 10 | Chinese Pharmacopoeia (2020 Edition, Part I), pp. 23–24 |
| 3 | *Paeonia lactiflora* Pall. | Paeoniaceae | Root | 9 | Chinese Pharmacopoeia (2020 Edition, Part I), pp. 108–109 |
| 4 | *Astragalus mongholicus* Bunge | Fabaceae | Root | 15 | Chinese Pharmacopoeia (2020 Edition, Part I), pp. 34–35 |
| 5 | Dioscorea opposita Thunb. | Dioscoreaceae | Rhizome | 20 | Chinese Pharmacopoeia (2020 Edition, Part I), p. 30 |
| 6 | Bupleurum chinense DC. | Apiaceae | Root | 9 | Chinese Pharmacopoeia (2020 Edition, Part I), p. 293 |
| 7 | *Acorus verus (L.)* Raf. | Acoraceae | Rhizome | 6 | Chinese Pharmacopoeia (2020 Edition, Part I), pp. 93–94 |
| 8 | Glycyrrhiza uralensis Fisch. ex DC. | Fabaceae | Root and rhizome | 6 | Chinese Pharmacopoeia (2020 Edition, Part I), pp. 88–89 |

**1 Information on the botanical drugs and extraction methods of CJG**

**1.1 Preparation of Test Samples**

**1.1.1 Preparation of HRMS Test Samples**

Aqueous extract: Accurately weigh 0.2 g of the mixed granules and add threefold volume of purified water. Extract by ultrasonication for 20 min, followed by centrifugation at 12,000 × g for 10 min. Collect the supernatant, filter through a 0.22 μm microporous membrane, and store at 4 °C until use.

Ethyl acetate fraction: Take 0.2 mL of the above aqueous extract and add an equal volume of ethyl acetate. Vortex for extraction, then centrifuge at 12,000 × g for 10 min. Collect the ethyl acetate layer, evaporate to dryness under nitrogen, reconstitute in 0.4 mL methanol, centrifuge again at 12,000 × g for 10 min, and collect the supernatant for storage at 4 °C.

Preparation of mixed standard solution: Appropriate amounts of rutin, saikosaponin B2, albiflorin, liquiritigenin, liquiritin, glycyrrhizic acid, paeonol, hyperoside, and astragaloside IV reference standards were dissolved in methanol to prepare stock solutions at 2 mg/mL. Equal volumes of each stock solution were mixed and diluted with methanol to obtain a mixed standard solution with each analyte at a final concentration of 50 μg/mL.

**1.1.2 Preparation of Samples for MRM Quantification**

Appropriate amounts of saikosaponin B2, hyperoside, liquiritin, albiflorin, and astragaloside IV reference standards were dissolved in methanol to prepare stock solutions at 2 mg/mL. The stock solutions were further mixed and serially diluted with methanol to obtain a series of working standard solutions for injection. The linear range for quantification of each analyte was 0.1–5 μg/mL.

Accurately weigh 0.2 g of the mixed granules and add sixfold volume of methanol. Extract by ultrasonication for 20 min, followed by centrifugation at 12,000 × g for 10 min. The supernatant was diluted 10-fold, 50-fold, and 500-fold with methanol, filtered through a 0.22 μm microporous membrane, and stored at 4 °C until analysis.

**1.2 Product Information of Granules**

The formula granules were purchased from the pharmacy of Xiyuan Hospital, China Academy of Chinese Medical Sciences. Manufacturer: Beijing Kangrentang Pharmaceutical Co., Ltd. (Address: No. 5 Niuhui Street, Niulanshan Town, Shunyi District, Beijing, China). Batch number: D1022-RD09-01. Date of manufacture: June 23, 2022. Expiration date: June 22, 2025. Storage conditions: Store at room temperature in a ventilated and light-protected environment.

**1.3 Reference Standards Information**

Information on the reference standards used in this study is provided in Table S3.

Table S3. Information of Reference Standards

| No. | Compound Name | CAS No. | Purity (%) | Supplier | Batch No. | Application |
| --- | --- | --- | --- | --- | --- | --- |
| 1 | Saikosaponin B2 | 58316-41-9 | ≥98% | Chengdu Ruifensi Biotechnology Co., Ltd. | RFS-C05102004003 | HRMS + MRM |
| 2 | Hyperoside | 482-36-0 | ≥98% | ChemFaces | CFS202104 | HRMS + MRM |
| 3 | Liquiritin | 551-15-5 | ≥98% | Shanghai Yuanye Biotechnology Co., Ltd. | P25J7F18328 | HRMS + MRM |
| 4 | Albiflorin | 39011-90-0 | ≥98% | ChemFaces | CFS202102 | HRMS + MRM |
| 5 | Astragaloside IV | 84687-43-4 | ≥98% | ChemFaces | CFS202102 | HRMS + MRM |
| 6 | Rutin | 153-18-4 | ≥98% | Chengdu Ruifensi Biotechnology Co., Ltd. | RFS-L00102202015 | HRMS only |
| 7 | Liquiritigenin | 578-86-9 | ≥98% | Shanghai Yuanye Biotechnology Co., Ltd. | ZM0314BD14 | HRMS only |
| 8 | Glycyrrhizic acid | 1405-86-3 | ≥98% | Shanghai Yuanye Biotechnology Co., Ltd. | P04N7F24311 | HRMS only |
| 9 | Paeonol | 552-41-0 | ≥98% | Chengdu Ruifensi Biotechnology Co., Ltd. | RFS-D00211812016 | HRMS only |

**2 Chemical Fingerprinting by High-Resolution Liquid Chromatography–Mass Spectrometry (LC–HRMS)**

**2.1 Instruments and Materials**

An integrated platform consisting of a Waters Acquity UPLC system coupled with a Synapt G2-Si HDMS high-resolution ion mobility mass spectrometer (Waters Corporation, USA) was employed. Chromatographic separation was performed on an Acquity UPLC BEH C18 column (2.1 mm × 100 mm, 1.7 μm, Waters). The column temperature was maintained at 35 °C, and the autosampler temperature was set at 4 °C. Methanol (HPLC grade), acetonitrile (HPLC grade), and formic acid (HPLC grade) were purchased from Fisher Scientific.

**2.2 Liquid Chromatographic and Mass Spectrometric Conditions**

LC conditions: The mobile phase consisted of solvent A (0.1% formic acid in water) and solvent B (0.1% formic acid in acetonitrile). The flow rate was 0.3 mL/min, and the injection volume was 2.0 μL. Two gradient elution programs were used in this study.

30-min gradient program: 0–0.8 min, 95% A; 0.8–5.0 min, 95%–88% A; 5.0–9.0 min, 88%–80% A; 9.0–11.0 min, 80%–75% A; 11.0–18.0 min, 75%–55% A; 18.0–25.0 min, 55%–1% A; 25.0–28.0 min, 1% A; 28.1–30.0 min, 95% A.

17-min gradient program: 0–1.0 min, 95% A; 1.0–12.0 min, 95%–0% A; 12.0–15.0 min, 0% A; 15.1–17.0 min, 95% A.

MS conditions: Data were acquired in both positive and negative electrospray ionization (ESI) modes. The capillary voltage was set at 3.5 kV (positive mode) and 2.2 kV (negative mode). The desolvation temperature was 450 °C (positive mode) and 400 °C (negative mode), with a source temperature of 110 °C. The desolvation gas flow was 800 L/h. The scan range was m/z 50–1200. The collision energy (CE) was set at 30–60 V. Sodium formate solution was used for mass calibration. Data acquisition and processing were performed using MassLynx V4.1 software (Waters).

**2.3 Identification Results**

Characteristic constituents were identified by comparing retention times and accurate molecular masses between reference standards and sample components. A total of 27 characteristic peaks were identified, among which 8 were confirmed using reference standards. The results are shown in Table S4 and Figures S1–S4.

Table S4. Information on Characteristic Components Identified by LC–HRMS

| No. | Name | Exact Mass | Observed m/z | Molecular Formula | RT (17 min) (min) | RT (30 min) (min) | Adduct Ion | Herbal Source |
| --- | --- | --- | --- | --- | --- | --- | --- | --- |
| 1 | 1,1-Kestotetraose | 666.22186 | 665.2145 | C24H42O21 | 0.83 | 0.86 | [M−H]⁻ | *Morinda officinalis* |
| 2 | Gallic acid | 170.02152 | 169.0143 | C7H6O5 | 1.54 | 1.54 | [M−H]⁻ | *Paeonia lactiflora* |
| 3 | Tryptophan | 204.08988 | 205.0973 | C11H12N2O2 | 3.06 | 3.69 | [M+H]⁺ | *Astragalus membranaceus*, Bupleurum chinense |
| 4 | Cianidanol | 290.07904 | 289.0722 | C15H14O6 | 3.33 | 4.63 | [M−H]⁻ | *Paeonia lactiflora* |
| 5 | Albiflorin | 480.1632 | 525.1623 | C23H28O11 | 3.9 | 6.86 | [M+FA−H]⁻ | *Paeonia lactiflora* |
| 6 | Paeoniflorin | 480.1632 | 525.1623 | C23H28O11 | 4.06 | 7.5 | [M+FA−H]⁻ | *Paeonia lactiflora* |
| 7 | Rutin | 610.15338 | 609.1469 | C27H30O16 | 4.12 | 8.58 | [M−H]⁻ | *Astragalus membranaceus*, Bupleurum chinense |
| 8 | Hyperoside | 464.09548 | 463.0881 | C21H20O12 | 4.22 | 8.69 | [M−H]⁻ | *Hypericum perforatum* |
| 9 | Liquiritin | 418.12638 | 417.119 | C21H22O9 | 4.32 | 8.74 | [M−H]⁻ | *Glycyrrhiza uralensis* |
| 10 | Isoliquiritin | 418.12638 | 417.118 | C21H22O9 | 5.09 | 11.73 | [M−H]⁻ | *Glycyrrhiza uralensis* |
| 11 | Formononetin-7-glucoside | 430.12638 | 431.1339 | C22H22O9 | 5.24 | 12.01 | [M+H]⁺ | *Astragalus membranaceus* |
| 12 | Liquiritigenin | 256.07356 | 257.0814 | C15H12O4 | 5.46 | 12.22 | [M+H]⁺ | *Glycyrrhiza uralensis* |
| 13 | Calycosin | 284.06847 | 285.0761 | C16H12O5 | 5.61 | 12.92 | [M+H]⁺ | *Astragalus membranaceus* |
| 14 | Astragaloside IV | 784.46091 | 829.4594 | C41H68O14 | 6.56 | 16.79 | [M+FA−H]⁻ | *Astragalus membranaceus* |
| 15 | Glycyrrhizic acid | 822.40379 | 823.4044 | C42H62O16 | 6.67 | 17.18 | [M+H]⁺ | *Glycyrrhiza uralensis* |
| 16 | Paeonol | 166.06299 | 167.07079 | C9H10O3 | 6.79 | 14.91 | [M+H]⁺ | *Paeonia lactiflora* |
| 17 | Formononetin | 268.07356 | 269.0813 | C16H12O4 | 6.8 | 16.15 | [M+H]⁺ | *Astragalus membranaceus* |
| 18 | Astragaloside II | 826.47147 | 871.4718 | C43H70O15 | 7.01 | 17.89 | [M+FA−H]⁻ | *Astragalus membranaceus* |
| 19 | Saikosaponin B2 | 780.46599 | 825.464 | C42H68O13 | 7.18 | 18.36 | [M+FA−H]⁻ | *Bupleurum chinense* |
| 20 | Saikosaponin A | 780.46599 | 825.4643 | C42H68O13 | 7.23 | 18.42 | [M+FA−H]⁻ | *Bupleurum chinense* |
| 21 | Saikosaponin B1 | 780.46599 | 825.4632 | C42H68O13 | 7.52 | 19.24 | [M+FA−H]⁻ | *Bupleurum chinense* |
| 22 | Astragaloside I | 868.48204 | 913.4825 | C45H72O16 | 7.83 | 19.78 | [M+FA−H]⁻ | *Astragalus membranaceus* |
| 23 | β-Asarone | 208.10994 | 209.1182 | C12H16O3 | 8 | 18.84 | [M+H]⁺ | *Acorus tatarinowii* |
| 24 | Isoastragaloside I | 868.48204 | 913.4823 | C45H72O16 | 8.12 | 20.26 | [M+FA−H]⁻ | *Astragalus membranaceus* |
| 25 | Saikosaponin D | 780.46599 | 825.4637 | C42H68O13 | 8.2 | 20.41 | [M+FA−H]⁻ | *Bupleurum chinense* |
| 26 | Glycyrrhetinic acid | 470.33961 | 471.35 | C30H46O4 | 10.16 | 22.69 | [M+H]⁺ | *Glycyrrhiza uralensis* |
| 27 | Furohyperforin | 552.3814 | 553.385 | C35H52O5 | 12.8 | 25.74 | [M+H]⁺ | *Hypericum perforatum* |

**2.4 Fingerprint Chromatograms of the Aqueous Extract**

**2.4.1 Base Peak Chromatograms (30-min Method)**

The base peak chromatograms (BPCs) of the aqueous extract and mixed reference standards in both positive and negative ion modes are shown in Figure S1.

Figure S1. Base peak chromatograms of the aqueous extract (30-min gradient). (A) Positive ion mode—sample; (B) Positive ion mode—mixed reference standards; (C) Negative ion mode—sample; (D) Negative ion mode—mixed reference standards.
(Reference standards: 5, Albiflorin; 7, Rutin; 8, Hyperoside; 9, Liquiritin; 12, Liquiritigenin; 14, Astragaloside IV; 15, Glycyrrhizic acid; 16, Paeonol; 19, Saikosaponin B2; 20, Saikosaponin A)

**2.4.2 Base Peak Chromatograms (17-min Method)**

Figure S2. Base peak chromatograms of the aqueous extract (17-min gradient). (A) Positive ion mode—sample; (B) Positive ion mode—mixed reference standards; (C) Negative ion mode—sample; (D) Negative ion mode—mixed reference standards.
(Reference standards: 5, Albiflorin; 7, Rutin; 8, Hyperoside; 9, Liquiritin; 12, Liquiritigenin; 14, Astragaloside IV; 15, Glycyrrhizic acid; 16, Paeonol; 19, Saikosaponin B2; 20, Saikosaponin A)

**2.5 Fingerprint Chromatograms of the Ethyl Acetate Fraction**

**2.5.1 Base Peak Chromatograms (30-min Method)**

Figure S3. Base peak chromatograms of the ethyl acetate fraction (30-min gradient). (A) Positive ion mode—sample; (B) Positive ion mode—mixed reference standards; (C) Negative ion mode—sample; (D) Negative ion mode—mixed reference standards.
(Reference standards: 5, Albiflorin; 7, Rutin; 8, Hyperoside; 9, Liquiritin; 12, Liquiritigenin; 14, Astragaloside IV; 15, Glycyrrhizic acid; 16, Paeonol; 19, Saikosaponin B2; 20, Saikosaponin A)

**2.5.2 Base Peak Chromatograms (17-min Method)**

Figure S4. Base peak chromatograms of the ethyl acetate fraction (17-min gradient). (A) Positive ion mode—sample; (B) Positive ion mode—mixed reference standards; (C) Negative ion mode—sample; (D) Negative ion mode—mixed reference standards.
(Reference standards: 5, Albiflorin; 7, Rutin; 8, Hyperoside; 9, Liquiritin; 12, Liquiritigenin; 14, Astragaloside IV; 15, Glycyrrhizic acid; 16, Paeonol; 19, Saikosaponin B2; 20, Saikosaponin A)

**2.6 Comparison of Fingerprint Chromatograms Obtained by Different Extraction Methods**
The chemical profiles of the aqueous extract and the ethyl acetate fraction were compared to evaluate the coverage of constituents with different polarity ranges. As shown in Figure S5, the aqueous extract was enriched in highly polar compounds, such as amino acids and organic acids, whereas the ethyl acetate fraction was enriched in moderately to weakly polar compounds, such as long-chain fatty acids and sapogenins.

Figure S5. Comparison of BPC fingerprint chromatograms of the aqueous extract and ethyl acetate fraction. (A) Positive ion mode; (B) Negative ion mode.

**3 Targeted Quantification by Triple Quadrupole MRM**

**3.1 Instruments and Conditions**

Quantitative analysis was performed using a SCIEX 5500 triple quadrupole mass spectrometer (SCIEX, USA) coupled with a Waters Acquity UPLC system (Waters Corporation, USA). Methanol (HPLC grade), acetonitrile (HPLC grade), and formic acid (HPLC grade) were purchased from Fisher Scientific.

**3.2 Liquid Chromatographic and Mass Spectrometric Conditions**

Chromatographic conditions: Separation was achieved on a Kinetex EVO C18 column (2.1 mm × 100 mm, 2.6 μm; Phenomenex). The mobile phase consisted of A (0.1% formic acid in water) and B (acetonitrile). The flow rate was 0.4 mL/min, and the injection volume was 2.0 μL. The column temperature was maintained at 45 °C, and the autosampler temperature was set at 4 °C. The gradient elution program was as follows: 0–1.5 min, 78%–67% A; 1.5–6.0 min, 67%–35% A; 6.0–6.9 min, 35%–1% A; 6.9–7.5 min, 1% A; 7.5–7.6 min, 1%–78% A; 7.6–8.0 min, 78% A.

Mass spectrometric conditions: An electrospray ionization (ESI) source was operated in multiple reaction monitoring (MRM) mode with simultaneous acquisition in both positive and negative ion modes. The ion spray voltage was set at 5500 V (positive mode) and 4500 V (negative mode). Curtain gas (CUR) was 20 psi; source temperature was 500 °C; nebulizer gas (GS1) and auxiliary heater gas (GS2) were both set at 50 psi. Data acquisition and processing were performed using Analyst 1.6.2 software (SCIEX). The MRM transitions and optimized parameters for the five quantified analytes are listed in Table S5.

Table S5. MRM Transitions and Optimized Mass Spectrometric Parameters

| No. | English Name | Precursor Ion (m/z) | Product Ion (m/z) | Declustering Potential (V) | Collision Energy (eV) | Ion Mode | Retention Time (min) |
| --- | --- | --- | --- | --- | --- | --- | --- |
| 1 | Saikosaponin B2 | 779.6 | 617.4 | -160 | -46 | ESI− | 3.63 |
| 2 | Hyperoside | 463.0 | 300.0 | -160 | -35 | ESI− | 2.40 |
| 3 | Liquiritin | 417.2 | 255.0 | -160 | -25 | ESI− | 2.38 |
| 4 | Albiflorin | 481.4 | 197.2 | 150 | 17 | ESI+ | 2.13 |
| 5 | Astragaloside IV | 785.5 | 143.3 | 135 | 15 | ESI+ | 3.35 |

**3.3 Method Validation**

**3.3.1 Linearity, Limit of Detection (LOD), and Limit of Quantification (LOQ)**

A series of mixed reference standard solutions (six concentration levels) were prepared. Calibration curves were constructed by plotting peak area (y) versus concentration (x, ng/mL) using linear regression. The LOD and LOQ were determined at signal-to-noise ratios (S/N) of ≥3 and ≥10, respectively. The results are summarized in Table S6.

Table S6. Linearity of Five Quantified Analytes

| **No.** | **Compound** | **Linear Range** | **Regression Equation** | **r²** |
| --- | --- | --- | --- | --- |
| 1 | Saikosaponin B2 | 0.1–5 μg/mL | y = 1.08×10³x + 4.17×10⁴ | 0.9974 |
| 2 | Hyperoside | 0.1–5 μg/mL | y = 8.70×10³x + 1.59×10⁵ | 0.9976 |
| 3 | Liquiritin | 0.1–5 μg/mL | y = 9.09×10³x + 4.46×10⁵ | 0.9976 |
| 4 | Albiflorin | 0.1–5 μg/mL | y = 2.49×10³x + 2.24×10⁴ | 0.9988 |
| 5 | Astragaloside IV | 0.1–5 μg/mL | y = 7.11×10²x + 2.51×10⁴ | 0.9994 |

**3.2.2 Precision and Accuracy**

Intra-day precision was evaluated using mixed reference standard solutions at three quality control (QC) levels: low (LQC, 0.25 μg/mL), medium (MQC, 2.5 μg/mL), and high (HQC, 4 μg/mL). Each level was analyzed six consecutive times within one day. Precision was expressed as the relative standard deviation (RSD) of peak areas. Six parallel samples were prepared for each QC level, and concentrations were calculated based on the calibration curves. Accuracy was expressed as the relative error (RE) between measured and nominal concentrations. The results are presented in Table S7.

Table S7. Intra-day Precision and Accuracy of Five Quantified Analytes

| No. | Compound | QC Level | Nominal (μg/mL) | Measured (mean ± SD, μg/mL) | Accuracy (RE, %) | Precision (RSD, %) |
| --- | --- | --- | --- | --- | --- | --- |
| 1 | Saikosaponin B2 | LQC | 0.25 | 0.247 ± 0.009 | -1.1 | 3.7 |
|  |  | MQC | 2.50 | 2.474 ± 0.063 | -1.0 | 2.6 |
|  |  | HQC | 4.00 | 3.969 ± 0.107 | -0.8 | 2.7 |
| 2 | Hyperoside | LQC | 0.25 | 0.247 ± 0.009 | -1.3 | 3.6 |
|  |  | MQC | 2.50 | 2.474 ± 0.041 | -1.1 | 1.7 |
|  |  | HQC | 4.00 | 3.996 ± 0.143 | -0.1 | 3.6 |
| 3 | Liquiritin | LQC | 0.25 | 0.253 ± 0.006 | 1.2 | 2.5 |
|  |  | MQC | 2.50 | 2.519 ± 0.065 | 0.8 | 2.6 |
|  |  | HQC | 4.00 | 3.906 ± 0.116 | -2.4 | 3.0 |
| 4 | Albiflorin | LQC | 0.25 | 0.253 ± 0.006 | 1.1 | 2.4 |
|  |  | MQC | 2.50 | 2.490 ± 0.078 | -0.4 | 3.1 |
|  |  | HQC | 4.00 | 4.051 ± 0.093 | 1.3 | 2.3 |
| 5 | Astragaloside | LQC | 0.25 | 0.249 ± 0.012 | -0.3 | 4.9 |
|  |  | MQC | 2.50 | 2.490 ± 0.087 | -0.4 | 3.5 |
|  |  | HQC | 4.00 | 3.998 ± 0.084 | -0.0 | 2.1 |

**3.3 Quantitative Results**

The contents of five characteristic constituents in the compound preparation are presented in Table S8.

Table S8. Contents of Characteristic Constituents in the Compound Preparation

| No. | Compound | Content in Granules | Selection Rationale |
| --- | --- | --- | --- |
| 1 | Saikosaponin B2 | 114.0 μg/g | Characteristic triterpenoid saponin from vinegar-processed Bupleurum chinense |
| 2 | Hyperoside | 67.2 μg/g | Major flavonoid from Hypericum perforatum and pharmacopoeial marker compound |
| 3 | Liquiritin | 792.0 μg/g | Major flavonoid from honey-fried Glycyrrhiza uralensis and pharmacopoeial marker compound |
| 4 | Albiflorin | 3.60 mg/g | Major monoterpene glycoside from Paeonia lactiflora |
| 5 | Astragaloside IV | 42.3 μg/g | Characteristic saponin from honey-fried Astragalus membranaceus and pharmacopoeial marker compound |

**3.4 MRM Chromatograms**

The MRM chromatograms of the blank solution, mixed reference standard solution, and sample solution for the five quantified analytes are shown in Figure S6. No significant interfering peaks were observed at the corresponding retention times in the blank chromatograms.

Figure S6. MRM chromatograms of five quantified analytes. (A) Blank control; (B) mixed reference standard solution (C4, 1000 ng/mL); (C) sample solution. From top to bottom: saikosaponin B2 (m/z 779.6→617.4), hyperoside (m/z 463.0→300.0), liquiritin (m/z 417.2→255.0), albiflorin (m/z 481.4→197.2), and astragaloside IV (m/z 785.5→143.3).

**Additional file 3**

**The other secondary outcomes**

**1 Total symptom score**

Compared with baseline, TCM symptom scores were significantly reduced in both groups at Week 4 (experimental group: from 16.07 ± 2.86 to 8.87 ± 2.40, a reduction of 7.20 ± 2.80; control group: from 15.31 ± 3.83 to 13.36 ± 3.35, a reduction of 1.96 ± 2.56; both P < 0.05). The reduction was significantly greater in the experimental group than in the control group from Week 2 to Week 4 (P < 0.05).

Figure S7. the total symptom score in Study. * *P* < 0.05, #*P* < 0.05.
